# Rationale and methods of the MOVI-HIIT! cluster-randomized controlled trial: an avatar-guided virtual platform for classroom activity breaks and its impact on cognition, adiposity, and fitness in preschoolers

**DOI:** 10.3389/fdgth.2026.1829401

**Published:** 2026-05-08

**Authors:** Fernanda Henriquez-Maquehue, Ana Díez-Fernández, Abel Ruiz-Hermosa, Alberto Bermejo-Cantarero, Óscar Navarro-Martínez, Beatriz Rodríguez-Martín, Mairena Sánchez-López

**Affiliations:** 1 Social and Health Care Research Center, Universidad de Castilla-La Mancha, Cuenca, Spain; 2 Faculty of Health and Social Sciences, Universidad de las Américas, Santiago, Chile; 3 Faculty of Nursing, Universidad de Castilla-La Mancha, Cuenca, Spain; 4 Faculty of Education, Universidad de Castilla-La Mancha, Ciudad Real, Spain; 5 Faculty of Nursing, Universidad de Castilla-La Mancha, Ciudad Real, Spain; 6 Department of Nursing, Physiotherapy and Occupational Therapy, Faculty of Health Sciences, University of Castilla-La Mancha, Talavera de La Reina, Spain

**Keywords:** body composition, classroom-based physical activity, cognition, early childhood education, high-intensity interval training, physical fitness

## Abstract

**Introduction:**

Classroom-based active breaks (ABs) have been shown to reduce sedentary time and increase physical activity in primary school children; however, evidence regarding their effects on body composition, physical fitness, cognition and other health-related outcomes remains limited in preschool children. This article describes the rationale and prespecified methods of the MOVI-HIIT study, a cluster-randomised controlled trial (RCT) evaluating a classroom-based, HIIT-inspired ABs intervention delivered through a gamified digital platform to improve body composition, physical fitness, and cognition in children aged 4–6 years. In addition, we outline a nested qualitative study designed to explore teachers’ perceptions of barriers and facilitators to programme implementation and monitoring.

**Methods:**

A RCT was conducted in nine schools in the province of Ciudad Real, Spain. Schools were randomly assigned to the MOVI-HIIT intervention group (*n* = 5) or the control group (*n* = 4). A total of 522 children aged 4–6 years were assessed at baseline (September 2022) and post-intervention (June 2023). The programme included two daily 6-minute HIIT-based ABs (20″/10″ work-to-rest ratio) guided by interactive avatars via a gamified digital platform. The primary outcomes were body composition, physical fitness, and executive function. Secondary outcomes included physical activity, sleep quality, blood pressure, and health-related quality of life. In parallel, a qualitative study using semi-structured interviews and focus groups examined teachers’ perceptions of barriers and facilitators related to the implementation and monitoring of the programme.

**Discussion:**

MOVI-HIIT represents the first intervention in early childhood education to integrate HIIT-based ABs with a gamified digital platform. Its structured, high-intensity exercise, combined with a mixed-methods process evaluation, may provide an innovative and potential reproducible model that can strengthen the evidence on classroom-based physical activity and contribute to creating active and healthy educational environments from early childhood onwards.

**Clinical Trial Registration:**

https://clinicaltrials.gov/study/NCT04863040, identifier NCT04863040.

## 1 Introduction

Sedentary behaviour together with low levels of physical activity (PA) among children are major public health concerns because they are linked to poorer health and developmental outcomes (1, 2). Evidence indicates that most European preschool-aged children fail to meet current international PA recommendations, and that their PA levels tend to decline progressively with age (3, 4). This is particularly worrying, as early childhood PA is crucial for healthy growth and the development of motor, cognitive, and social skills.

Schools play a fundamental role in shaping children's daily routines and health behaviour, making them an ideal setting for promoting PA (5). However, a systematic review that included studies conducted in several countries reported that children are insufficiently active during school hours, spending most of this time engaged in sedentary activities (6). Given that children spend a considerable portion of the day at school, implementing strategies to increase PA and reduce sedentary time within this environment is essential.

In recent years, active breaks (ABs) have gained attention as an effective classroom strategy to reduce sedentary behaviour and increase children's PA during the school day (7, 8). Defined as short periods of PA integrated into lessons, ABs aim to interrupt sedentary time without detracting from academic learning, and their viability and feasibility of implementation in school settings have been widely demonstrated (8, 9). Although most studies have focused on demonstrating that ABs are an effective strategy to increase children's PA levels, evidence on their impact in other outcomes remain limited (10, 11). The effects of ABs on physical fitness and body composition have been less explored, and current findings are heterogeneous and inconclusive, possibly due to methodological variability across interventions (10, 12–15). Similarly, although regular PA has been shown to enhance attention and cognitive processing through various neural mechanisms, evidence supporting these effects within classroom-based ABs remains scarce (12, 16, 17). Overall, these findings highlight the scarcity of methodologically robust studies examining the benefits of ABs, particularly in early childhood, an age group that has been scarcely addressed in this type of research (17).

In this context, exploring innovative approaches to enhance the effectiveness of ABs is particularly relevant. High-intensity interval training (HIIT) has emerged as a time-efficient and effective method to improve children's health and fitness, alternating short bouts of vigorous activity with brief recovery periods (18). However, although HIIT appears well suited to children's natural activity patterns (19), evidence on the effectiveness of HIIT interventions in children under 6 years of age remains limited. Classroom-integrated HIIT programmes have been scarcely studied in preschoolers, with insufficient evidence regarding their effects on physical fitness, body composition, and cognition. Therefore, developing an ABs programme grounded in HIIT principles could represent a promising approach to optimize its benefits in early childhood and to provide new evidence in this field.

On the other hand, integrating digital technologies into education can enhance students’ engagement and participation (20). When designed with interactive or gamified features, these tools, including screen-based resources, can encourage movement and support more active learning experiences (21). Therefore, exploring digital platforms to deliver HIIT-based ABs in preschool settings is particularly relevant, as to our knowledge no previous studies have implemented a gamified digital approach for this purpose.

The present study describes the rationale and prespecified methods of a cluster-randomized controlled trial (RCT) —the MOVI-HIIT study — aimed at: (i) evaluating the effectiveness of a classroom-based HIIT inspired AB intervention, delivered through a gamified digital platform, on preschool children's body composition, physical fitness, and executive function; and (ii) exploring teachers’ perceptions of barriers and facilitators to its implementation and monitoring through a nested qualitative study. The trial procedures and analyses are reported here to support transparency and reproducibility, in line with the purpose of a study protocol, while intervention outcomes will be reported separately.

## 2 Methods and analysis

This paper is reported in accordance with the SPIRIT statement (22) to enhance completeness and transparency of the prespecified trial methods. The intervention is described using the TIDieR checklist to enable replication (23). Reporting of the nested qualitative component will follow the COREQ checklist (24).

### 2.1 Design

This study protocol describes the MOVI-HIIT study, conducted as a two-arm RCT (ClinicalTrials.gov: NCT04863040), including an intervention group (IG) and a control group (CG). Ten schools from different municipalities in the province of Ciudad Real (Castilla-La Mancha region, Spain) were invited to participate. One school declined to participate, noting concerns about potential additional workload for teachers and limited digital skills; therefore, nine schools were included. After agreeing to take part, each school was randomly allocated to either the IG or CG using the StatsDirect statistical software (version 16). Five schools (two public rural, two public urban, and one private urban) were assigned to the IG, where the MOVI-HIIT programme was implemented, while four schools (two public rural and two public urban) were assigned to the CG, which continued usual school activities.

**Figure 1:**
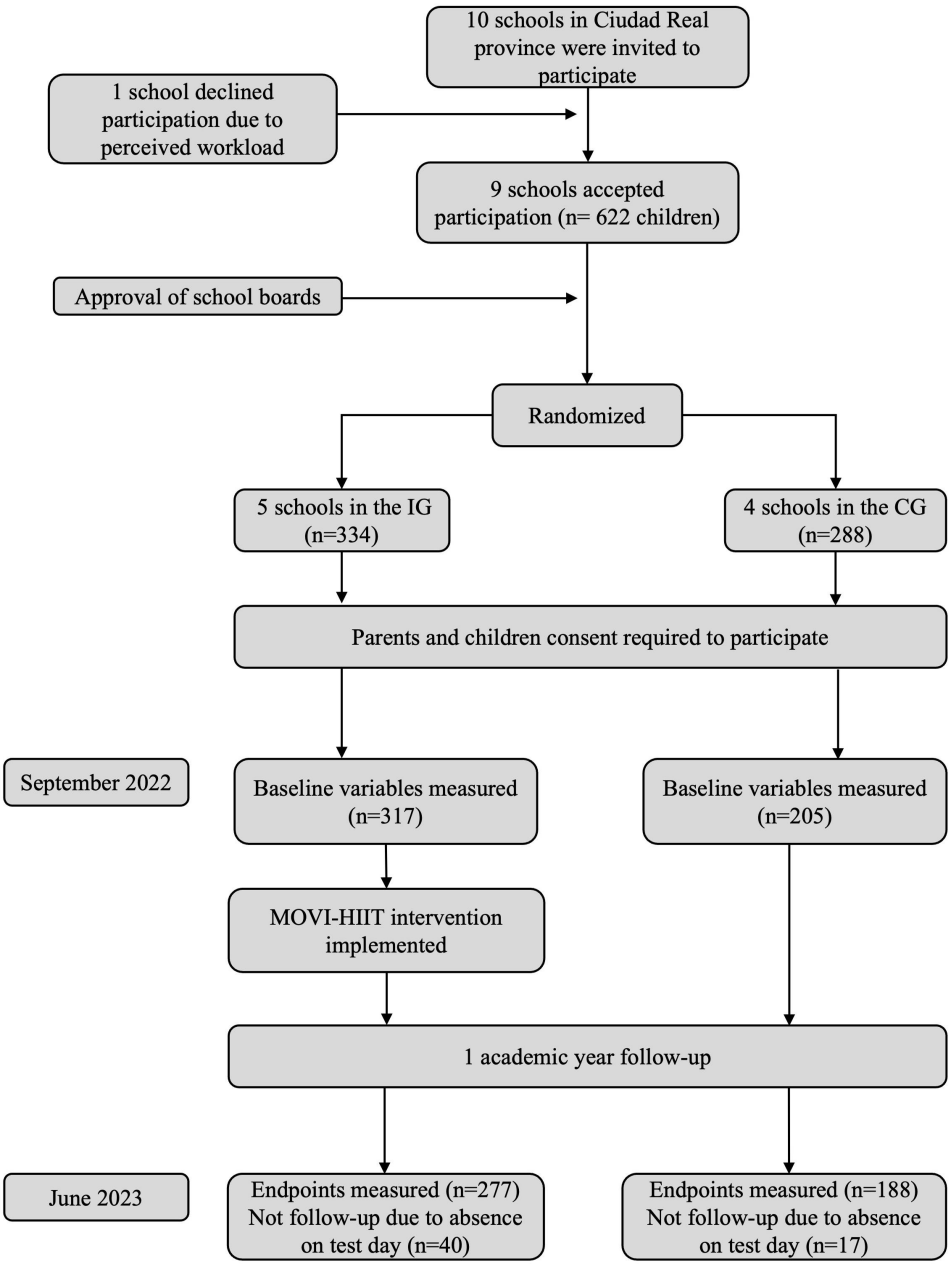
Flow diagram of participant recruitment and allocation (MOVI-HIIT trial) CG, control group; IG, intervention group.

 Figure 1 illustrates the flowchart of the participant recruitment process. The effectiveness of the classroom-integrated MOVI-HIIT intervention was assessed in both groups at baseline and post-intervention.

In addition, a nested qualitative study was conducted to explore teachers’ perceptions of the barriers and facilitators related to the implementation and monitoring of the intervention programme.

### 2.2 Participants and inclusion criteria

To participate in the study, schools had to be located in the province of Ciudad Real, have at least one classroom in each of the second and third years of preschool education (aged 4 to 6 years), and obtain approval from the School Board for the intervention and the baseline and post-intervention assessments.

Participating schoolchildren were required to be enrolled in the second or third year of preschool education, have no congenital or acquired conditions affecting language acquisition (oral or sign language), and not present any physical or mental disorder identified by parents or teachers that could hinder participation in physical activities. Additionally, children with chronic diseases such as cardiovascular disorders, diabetes, or asthma were excluded if participation was contraindicated, as determined by their pediatrician after reviewing the activity programme.

Since this was a classroom-based intervention, teachers integrated the ABs into the regular school schedule, meaning the programme was delivered to all children in the IG schools. However, only children whose parents or legal guardians provided written informed consent were included as study participants.

### 2.3 Intervention

#### 2.3.1 MOVI-HIIT! programme: description

The design of the MOVI-HIIT intervention was grounded in the socio-ecological model of behaviour change, addressing factors at the individual, family, and school levels (25). The programme was implemented over one academic year (October 2022 to May 2023) and consisted of two daily 6-minute classroom-based ABs involving physical exercise based on HIIT principles, following a 20"/10" work-to-rest ratio. ABs were delivered through a virtual platform (http://www.movihiit.es) guided by animated avatars, and required no specific equipment, as all classrooms were equipped with interactive screen and internet access.

**Figure 2:**
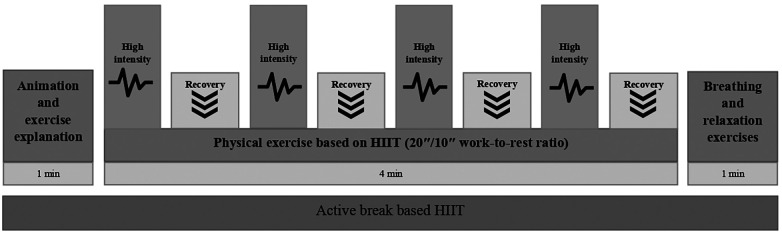
Session design MOVI-HIIT protocol (2 blocks alternating four exercises of 20 s of high-intensity activity at 85%–90% of maximum heart rate with 10 s of recovery at 65%–75%).

Each AB followed a structured format: approximately 1 min of animation and exercise explanation, 4 min of physical exercise based on HIIT, and 1 min of breathing and relaxation exercises to facilitate the transition back to academic tasks. The HIIT phase consisted of four exercises (e.g., squats, jumping jacks, or running in place), performed at high intensity (80%–90% of maximum heart rate) for 20 s, interspersed with 10-second recovery periods (65%–75% of maximum heart rate). The sequence of four exercises was repeated twice Figure 2.

**Figure 3:**
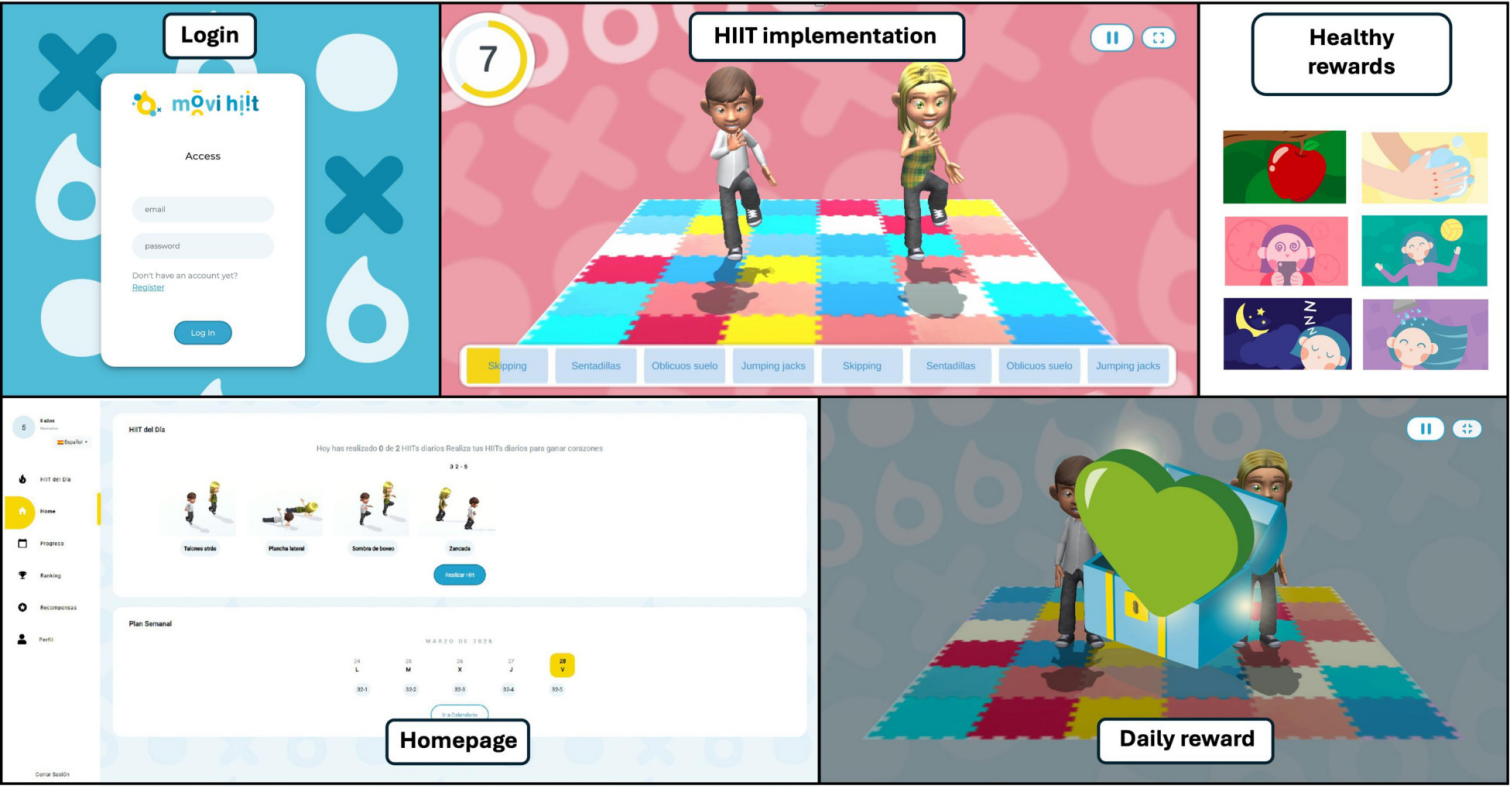
Visual structure platform.

To ensure feasibility and fidelity of implementation, a teacher-oriented virtual platform was designed to guide all ABs. Students were simply required to follow the avatars’ instructions and replicate their movements. The platform is gamified, featuring a ranking system, a weekly progress bar, and a system of daily, weekly, and monthly rewards designed to enhance children's motivation. Figure 3 shows the visual structure and interface of the platform.

The preschoolers in the CG received one 45-minute session per week of psychomotor or physical education and continued with their regular classroom activities. Teachers were asked not to implement any changes in their teaching practices during the study period. As an incentive, the research team committed to providing access and training to the MOVI-HIIT platform to CG teachers after the intervention.

#### 2.3.2 MOVI-HIIT! organization and delivery

The intervention programme was designed by graduates in Sport and Exercise Sciences, together with Physical Education teachers, and was implemented by the classroom tutors at each participating school. At the beginning of the intervention, teachers attended a 4-hour training workshop in which the research team presented the materials and the virtual platform and provided detailed guidance on their use. In addition, teachers were given a one-week familiarisation period with the programme under the supervision of the research team. To respect teachers’ autonomy and facilitate the integration of the intervention into classroom routines, teachers were allowed to choose the most suitable time of day to implement the HIIT breaks. However, team members recommended carrying them out when schoolchildren (i) had been sitting for a long time, (ii) were not paying attention, or (iii) seemed tired from academic tasks.

#### 2.3.3 Implementation and process evaluation

Implementation was evaluated using a mixed-methods process evaluation, including platform-derived delivery metrics, objective checks of exercise intensity and acceptability, and a nested qualitative study exploring teachers’ perceived barriers and facilitators.

##### 2.3.3.1 Intervention fidelity and adherence

Prior to implementation, a pilot with heart-rate monitors (Polar A300 watch and H7 chest strap) was conducted to confirm that the HIIT breaks elicited the desired exercise intensity. In week 12, intensity was reassessed *in situ* by fitting heart rate monitors to a random sample of children (*n* = 92) from three intervention schools during the ABs. Programme acceptability was assessed on the same day using a one-item enjoyment scale adapted from the Smileyometer (“How much fun did you have today during the active breaks?”; response options: not at all, somewhat, very much; scored 1–3) (26).

Thus, the process evaluation included indicators of fidelity (exercise intensity), acceptability (children’s enjoyment), delivered dose, and feasibility/ barriers to implementation.

Intervention dose (delivered dose) was tracked by the platform, which automatically logged the number of ABs sessions per class on a weekly and monthly basis and displayed these counts to teachers and students as real-time feedback. When monthly counts were low, the research team conducted follow-up visits with the corresponding teachers to identify implementation barriers and provide specific recommendations.

 When implementation or technical problems were detected, the research team also provided troubleshooting support through the communication channel already established with teachers and, when needed, additional follow-up visits.

Ongoing engagement was supported through a dedicated communication channel (phone number and e-mail) available to teachers throughout the school year, monthly check-ins, and two in-person meetings with classroom teachers (at baseline and after four months). To acknowledge participation and foster motivation, children received small programme-branded incentives (e.g., stickers, drawstring bags, or sports equipment).

After two months of implementation, teachers completed a questionnaire regarding the frequency of HIIT breaks per week, students’ attitudes and behaviour during sessions, perceived barriers to implementation, and suggestions for programme improvement. This information was used to document feasibility and to guide corrective actions during programme delivery.

##### 2.3.3.2 Barriers and facilitators to implementation

To understand teachers’ perceptions of the barriers and facilitators to the implementation and monitoring of the intervention, a qualitative study was conducted based on Bronfenbrenner’s socio-ecological model, which conceptualises human behaviour as multifactorial and dynamic (27).

For data collection, semi-structured interviews and focus groups were used, depending on the type of participant. In the case of classroom teachers from participating schools, both methods were applied to explore their knowledge, beliefs, and perceptions regarding the barriers and facilitators to the implementation of the MOVI-HIIT intervention in the classroom.

To gain a deeper understanding of the phenomenon, a purposive convenience sampling strategy with maximum variation was used. Participants were selected from teachers who had implemented the intervention and met the prespecified inclusion criteria. To ensure maximum variation in perspectives, the sample included teachers with different sociodemographic characteristics (sex and age), educational levels (Bachelor's Master's, or Doctorate), professional experience (<10 years, 10–20 years, and >20 years), and school characteristics (public or state-funded; urban or rural), as well as different professional roles (classroom teacher, coordinator, or member of the school leadership team).

The interviews and focus groups were conducted by two researchers trained in qualitative methods. Each session lasted 30–60 min, was video recorded with prior consent, and was conducted via Microsoft Teams.

Transcripts were analysed using thematic analysis. Two researchers independently coded the data and developed themes through an interactive process, resolving discrepancies by discussion (and consultation with a third researcher when needed). Data were managed using ATLAS.ti. Trustworthiness was enhanced through triangulation across data sources (interviews and focus groups) and participant characteristics, and data collection continued until thematic saturation was reached, that is, the point at which additional interviews or focus groups no longer yielded new information or analytical themes relevant to teacher's perceptions across the main participant profiles represented in the sample.

### 2.4 Outcome measures

Baseline and post-intervention outcomes (Table 1) were assessed in both groups at two time points: at the beginning (September 2022) and at the end (June 2023) of the school year. All measurements were performed at the schools by trained investigators who were blinded to group allocation to reduce measurement bias and minimise inter-observer variability.

**Table 1:** Study variables.

Type of variable	Specific variables	Tools
Primary variables	Anthropometry: *weight, height, BMI*	Seca 861 and Seca 222
	Body composition: *waist circumference, body fat, fat-free mass*	Flexible measuring tape, Tanita® BC-418 MA 8-electrode
	Physical fitness: *cardiorespiratory fitness, muscular strength, speed-agility, and balance*	PREFIT battery
	Cognition: *inhibition, cognitive flexibility, working memory, general cognitive ability*	NIH Toolbox, Word Span Test, and BADyG-I
Secondary variables	PA, sedentary and sleep quality: *weekly PA, school-day PA, sedentary time, sleep period time, total sleep time, wake after sleep onset, number of awakenings, and sleep efficiency*	Axivity AX3 accelerometer and GENEActiv accelerometer
	Active commuting to/from school	Self-administered questionnaire
	Blood pressure	Omron® M5-I automatic monitor
	Health-related quality of life	Kiddy-KINDL questionnaire
Possible confounding variables	Age	Self-administered questionnaire
	Sex	
	Birth weight	
	Gestational age	
	Parental height and weight	
	Area of residence	
	Country of origin	
	Family socioeconomic status	Parental education and occupation questionnaire
	Food Consumption	Children’s Eating Habits Questionnaire
	Breastfeeding	IFPS questionnaires

BMI, body mass index; PA, physical activity.

#### 2.4.1 Primary outcome measures

##### 2.4.1.1 Anthropometric variables

Anthropometric measurements were taken twice, and the average value was used for the analysis. Weight was assessed with a calibrated scale (Seca 861) while the child was barefoot and wearing light clothing. Height was measured with a wall-mounted stadiometer (Seca 222), with the child barefoot, standing upright, and the midsagittal plane aligned against the stadiometer's backboard. Children were required to look straight ahead, with their line of vision parallel to the floor. Body mass index (BMI) was calculated from the mean weight and height values, using the standard formula: weight (kg)/height (m^2^).

##### 2.4.1.2 Body composition

Waist circumference was measured as the mean of three readings taken at the midpoint between the last rib and the iliac crest, at the end of a normal expiration, using a flexible measuring tape. Body fat percentage and fat-free mass were assessed using a Tanita® BC-418 MA 8-electrode bioelectrical impedance analyser.

##### 2.4.1.3 Physical fitness

Health-related physical fitness was assessed using the PREFIT battery (28) which included the following tests:

Cardiorespiratory fitness was assessed using the 20-m shuttle run test (Course Navette), validated to estimate maximal aerobic capacity in preschool children. Participants ran back and forth between two 20-m lines in time with audio signals, starting at 6.5 km·h⁻^1^ and increasing by 0.5 km·h⁻^1^ per minute until exhaustion. Maximal oxygen uptake (VO₂max) was estimated using the PREFIT formula (29).

Muscular strength was assessed using handgrip dynamometry (Takey®, TKK 5401 Grip-D) and the standing long jump test, evaluating upper- and lower-body explosive strength, respectively. In the handgrip test, participants squeezed the dynamometer continuously for 2 s with the right and left hands alternately, performing two trials per hand with short rest intervals; the maximum value (kg) was recorded. In the standing long jump, children jumped horizontally from a standing position, and the best of three attempts was recorded in centimetres (28).

Speed/agility was assessed using the 4 × 10 m shuttle run test, which measures movement speed, agility, and coordination. Children were instructed to run as fast as possible between two lines 10 m apart, completing four shuttles. Two trials were performed, with a 5-minute rest interval between attempts (28).

Balance was assessed using a 30-second one-leg stance test. Children stood on one leg for as long as possible, up to 30 s. Two trials were performed with each leg, and the best result was recorded (28).

##### 2.4.1.4 Cognition

Inhibition and cognitive flexibility were assessed using the NIH Toolbox Executive Function Battery, which includes the Flanker Inhibitory Control and Attention Test and the Dimensional Change Card Sort Test (DCCS) (30). In the Flanker task, children identified the left–right orientation of a central stimulus while ignoring flanking stimuli, which could be congruent or incongruent. In the DCCS, stimuli varying by color or shape were presented, and children were required to shift their responses according to the relevant sorting dimension. Both tasks included four practice trials, and a composite score was calculated for each (31).

Working memory was measured using the Word Span Test, which included forward and backward tasks (32). In the forward task, children repeated sequences of two to seven words in the same order, whereas in the backward task, they recalled them in reverse order. The test ended when the child failed both trials at the same sequences length, and a mean score was calculated.

General cognitive ability was assessed using two subtests from the Differential and General Aptitude Battery for Early Childhood Education (BADyG-I) (33): the Quantitative Concepts subtest, which evaluates basic numerical and arithmetic reasoning, and the Vocabulary subtest, which assesses verbal comprehension and general knowledge through image–word association tasks.

#### 2.4.2 Secondary outcomes measures

##### 2.4.2.1 Physical activity and sleep quality

Twenty-four-hour assessment. A randomly selected subsample of 250 children from both study groups wore an Axivity AX3 accelerometer (Axivity Ltd., UK) on the non-dominant wrist for six consecutive days, including weekends (February–May 2023). Devices were initialized at 100 Hz with a ± 8 g range to record raw triaxial acceleration in gravitational units (g) and data were processed in R using the GGIR package (version 3.0-0) (34). A valid day was defined as at least 10 h of awake wear, and participants provided a minimum of four valid days, including one weekend day. Sleep variables, including sleep period time, total sleep time, wake after sleep onset, number of awakenings, and sleep efficiency, were derived using GGIR algorithms based on sustained inactivity bouts, supported by sleep diaries or, when unavailable, the HDCZA algorithm (35). During waking hours, PA intensities (sedentary, light, moderate, vigorous) were determined using the cut-points proposed by Hildebrand et al. (36, 37).

School-day assessment. To assess PA during school hours, an independent subsample of 150 children from both study groups wore a GENEActiv Original accelerometer (ActivInsights, UK) on the non-dominant wrist from 09:00 to 14:00 during two measurement waves (September–November 2022 and April–May 2023). Devices were initialized at 30 Hz with a ± 8 g dynamic range and recorded raw triaxial acceleration in g. Raw accelerometer data were processed in R (version 4.3.2) using the GGIR package (version 3.0.0) (38), including and initial autocalibration step based on local gravity and temperature according to the algorithm described by van Hees et al. (38). Non-wear time was identified using the standard deviation and range of raw acceleration signals over 60-minute windows with 15-minute increments, following the default GGIR approach (39). Due to the short monitoring duration, physical activity was derived as ActiGraph-equivalent activity counts from raw acceleration using the actilifecounts package (version 1.1.1) (40), according to the method proposed by Neishabouri et al. (41), with a 5-second epoch length. Activity intensity was categorized as sedentary (<306 counts/5 s), light (306–817 counts/5 s), moderate (818–1968 counts/5 s), and vigorous (≥1969 counts/5 s) using the vector magnitude cut-points validated by Chandler et al. for wrist-worn accelerometers in children (42). The school day was analyzed between 09:30 and 13:30 to exclude arrival and departure times, and a valid school day was defined as at least 3 h of wear time within this period after exclusion of non-wear.

##### 2.4.2.2 Active commuting to/from school

The Mode and Frequency of Commuting to and From School Questionnaire was administered to parents. This questionnaire has been previously validated against accelerometry in Spanish children and adolescents (43, 44). It includes four items assessing both the usual mode and the weekly frequency of commuting between home and school. Specifically, parents reported the usual mode of commuting by answering the following questions: (1) How does your child usually get to school? and (2) How does your child usually get home from school? Weekly frequency of commuting modes was assessed using the questions: (3) How does your child get to school each day? and (4) How does your child get home from school each day? Response options for all items were: (a) walking; (b) cycling; (c) car; (d) motorcycle; (e) school bus; (f) public bus; (g) subway/train/tram; or (h) other (with specification required). Active commuting to and from school was defined as regular travel by walking or cycling, whereas inactive commuting included travel by car, motorcycle, school bus, public bus, subway, train, or tram. In addition, parents reported their child's distance and time to school. Distance was assessed by the question: (1) How far from the school does your child live?, with response options: (a) < 0.5 km; (b) 0.5 to <1.5 km; (c) 1.5 to <3 km; (d) 3 to <6 km; and (e) ≥ 6 km. Travel time was assessed using the question: (2) How long does it usually take your child to get to school from home?, with response options: (a) < 5 min; (b) 5 to <15 min; (c) 15 to <30 min; (d) 30 to <60 min; and (e) ≥ 60 min (45).

##### 2.4.2.3 Blood pressure

Blood pressure was measured twice, 5 min apart, after a minimum 5-minute rest period, with children seated in a quiet environment and the right arm slightly flexed at heart level. The mean of both readings was used for analysis. Measurements were obtained using an Omron® M5-I automatic monitor (Omron Healthcare UK Ltd.) with cuff sizes adjusted to arm circumference (46).

##### 2.4.2.4 Health-related quality of life

Was measured using the Kiddy-KINDL questionnaire, a validated Spanish version of the KINDL questionnaire for children aged 4 to 7 years (47). The questionnaire consists of 6 dimensions on a 5-point Likert scale, with a total of 24 items, exploring physical, emotional, social and school aspects of childreńs lives. Children and their parents were asked to complete this questionnaire separately to compare results.

#### 2.4.3 Confounding variables

The following possible confounding variables were assessed through a self-administered questionnaire completed by parents or legal guardians: sex, age, birth weight, gestational age, parental height and weight, area of residence (urban or rural), and country of origin. Additionally, other relevant variables were recorded:

Family socioeconomic status. Was assessed as in previous MOVI studies (48). Using a self-administered questionnaire on parental education and occupation. Education was classified into three levels: primary (no or incomplete primary education), secondary (primary or upper secondary education), and university (degree or doctorate). Occupation was grouped into five categories: (i) supervisor/manager or self-employed with ≥10 employees, (ii) supervisor/manager or self-employed with <10 employees, (iii) self-employed with no employees, (iv) unskilled worker, and (v) homemaker, unemployed, or other. A family socioeconomic status index combining both variables was then calculated, yielding five levels: lower, lower/middle, middle, upper middle and upper (49).

Food Consumption. The Spanish version of the validated questionnaire on children's eating habits was used for children aged 2 to 9 years. The questionnaire was completed by the parents (50).

Breastfeeding. Parents reported the type of feeding provided during the first 24 months of their child's life. For each month, mothers indicated whether the child received breast milk, formula, or both, and whether complementary foods were introduced. Based on this information, breastfeeding was classified into exclusive, mixed, or formula feeding, and the duration of each was recorded (51).

### 2.5 Data analysis

#### 2.5.1 Sample size

In a trial with multiple outcomes, the sample size was estimated using a prespecified reference outcome rather than calculating a separate sample size for each endpoint. Cardiorespiratory fitness (VO₂max, mL·kg⁻1·min⁻1) was selected as the reference outcome because it was one of the main study outcomes and the one for which the most robust assumptions were available from a previous comparable school-based cluster-randomized intervention conducted by our group (MOVI-2) (52). Based on that study, an effect size (Cohen’s d) of at least 0.2 was expected when comparing the IG with CG. However, since the study hypotheses focused on less physically fit schoolchildren—who might benefit more—an effect size of 0.3 was assumed.

The calculation was performed following the methods of Donner and Klar (53), using Optimal Design software, with the multi-site cluster randomized trials, treatment at level 2 option. An average of 40 children per school (cluster), a statistical power of 0.80, a significance level of 0.05, and an effect size of 0.3 were assumed. Under these conditions, a total sample size of 10 schools was estimated. An average of 40 children per school was considered under two assumptions: a) although the actual average was approximately 100 children per school, the main analysis focused on the 50% with the lowest baseline VO₂max levels by age and sex; and b) a dropout rate of 15% was anticipated. Although cognitive and adiposity outcomes were also assessed, the trial was not specifically powered for each individual endpoint; therefore, these results should be interpreted accordingly.

#### 2.5.2 Statistical analysis

The statistical analysis will have three phases. The first will consist of verifying that randomisation was effective in creating two comparable groups of schoolchildren, exploring the presence of extreme values and outliers, and assessing the degree of fit of the main variables to the normal distribution.

In the second phase, mixed regression models will be used, taking each outcome variable as the dependent variable, the intervention as a fixed effect (1 = IG and 0 = CG), adjusting for baseline values, age, sex, and including school (cluster) as a random effect. Given that this is a cluster-randomized trial, randomization is the main strategy to minimize selection bias, whereas covariate adjustment is intended primarily to improve precision and account for chance baseline imbalances. The results will be expressed as absolute differences in the changes in the variables between baseline and final measurements [95% confidence interval (95%CI)]. For binary outcomes (e.g., overweight/obesity), logistic regression models will be used, and odds ratios with their 95% CIs will be reported.

In the third phase, a sensitivity analysis will be performed to verify whether the results, using a different variable adjustment method, have the same direction and similar magnitude. To do this, the IG and CG will be compared using the propensity score statistical method to account for the imbalance of covariates in baseline measurements between clusters. The propensity score estimates the effect of the intervention using a causal inference model, explaining what would have happened if all subjects in the IG and CG had the same characteristics at the start of the study. Each subject is matched to a subject with similar characteristics using a caliper of 0.40 using the psmatch2 command.

All analyses will be conducted on an intention-to-treat basis, maintaining participants in their originally assigned IG or CG, regardless of the number of active-HIIT breaks they received, and considering the CONSORT recommendations for this specific type of cluster design (54). In addition to analyses for the total sample, subgroup analyses by age, sex, and socioeconomic status will be conducted.

However, to assess adherence to the intervention, data will be collected on the number of active HIIT breaks each group received. Children in classes that delivered ≥80% of the scheduled breaks will be considered to have received the intervention.

Results with a *p* < 0.05 will be considered statistically significant, and the analysis will be performed using the R and STATA18 statistical packages.

## 3 Discussion

This study was designed to address key challenges in promoting children's health: low levels of PA and high sedentary time among preschoolers, particularly during the school day (3, 4, 6). Although classroom-integrated PA has been associated with health and learning benefits, the evidence remains mixed and recent reviews highlight the need for more methodologically robust interventions, especially in preschool settings (10, 11, 17).

The MOVI-HIIT study was developed to help fill this gap by testing an AB–based HIIT intervention supported by a gamified digital platform. To our knowledge, it is the first programme of this kind in early childhood education to be evaluated in relation to body composition, physical fitness, and executive function. In addition, the nested qualitative study will explore teachers’ perceptions of barriers and facilitators to the implementation and monitoring, providing insights into real-world feasibility and scalability.

MOVI-HIIT extends previous AB research by incorporating a structured, high-intensity exercise, and digital delivery/monitoring component. Whereas most AB interventions in early childhood education have relied on unstructured or moderate-intensity activities (55, 56). MOVI-HIIT applies HIIT principles to brief sessions in a time-efficient format suited to preschoolers’ developmental characteristics. Previous studies implementing HIIT-based ABs programs in primary and secondary education have reported positive effects on physical fitness, body composition, and cognitive function (8, 57). However, to the best of our knowledge, no previous studies have implemented this type of intervention in early childhood education, and digital gamified delivery has rarely been examined in this context.

Therefore, the use of a gamified digital platform guided by interactive avatars represents a novel approach in this context, as it not only allows teachers to deliver sessions independently, supporting consistency, standardisation, and reproducibility across classrooms. In addition, the platform facilitates flexible integration of ABs within daily classroom routines. This technological integration aligns with emerging evidence that gamified digital tools can improve motivation, participation, and adherence in both educational and movement-based activities (20, 21).

Another strength of the MOVI-HIIT study lies in its rigorous methodological design. The RCT structure minimises contamination between groups while preserving the authenticity of the school environment, thereby enhancing external validity. Implementing the intervention within schools supports accessibility and scalability, as it can reach all children across diverse backgrounds. Moreover, previous evidence shows that increasing movement opportunities during the school day does not compromise academic performance, supporting the feasibility of integrating structured PA programmes into classroom routines (58). In addition, the use of validated and standardised instruments enhances measurement quality and comparability with previous studies. Finally, the nested qualitative component exploring teachers’ perceptions of barriers and facilitators will provide valuable insights into implementation processes, informing scalability and potential sustainability in real-world educational contexts.

Nevertheless, some limitations should be acknowledged. As with most behavioural interventions, blinding participants was not feasible, which may have introduced performance bias. Furthermore, variability in teacher motivation or classroom dynamics could affect programme fidelity; however, this was addressed through initial teacher training, ongoing technical support, and automated monitoring via the digital platform. Technical issues, such as temporary internet or power outages, could disrupt programme delivery; however, this risk was minimised by providing downloadable ABs and technical support staff from the research team. In addition, accelerometry in a subsample may limit the generalisability of findings related to PA and sleep, and data reported by parents may be subject to recall bias.

Additionally, although the trial included cognitive and adiposity outcomes, the sample size was estimated using VO_2_max as the reference outcome; therefore, the study may have had different statistical power across endpoints, particularly for outcomes with smaller expected effects.

Another limitation identified was that, although teachers were allowed to choose the timing of ABs to facilitate their implementation, the exact time of their implementation was not systematically recorded as a quantitative variable; therefore, possible differences according to the time of day could not be examined. Finally, the implementation of similar ABs in the control group was not formally monitored or objectively recorded; therefore, some degree of contamination could not be completely ruled out.

In conclusion, the MOVI-HIIT study presents an innovative and potentially scalable model for integrating HIIT-based ABs into preschool classrooms through a gamified digital platform. By detailing the prespecified methods and implementation strategy, this article aims to support transparency and reproducibility, and to inform future school-based interventions. The study will provide insights into the potential effects of HIIT-based ABs on body composition, fitness, and executive function, alongside teachers’ perspectives on implementation, which may help guide future educational practice and health policy to foster active and healthy school environments.

## 4 Ethics and dissemination

The Clinical Research Ethics Committee of the University Hospital of Ciudad Real approved the study protocol (registration number: REG:C-254). Following approval by the school directors and their Boards of Governors, parents received a letter requesting written informed consent for their child's participation and inviting them to an informational meeting about the study objectives and procedures. In addition, the children had to verbally express their willingness to participate in the baseline and final assessments. After each evaluation of the child's health status, parents were provided with a written report summarising their child's results, and appropriate recommendations were offered if any alteration were detected.

Although the PA intervention could not be blinded to participants, researchers conducting the measurements were blinded to group allocation. To ensure confidentiality and minimise bias, each participant was assigned an identification code, and personal data were stored separately from study data.
